# Oral‐bacterial‐induced arterial and venous thrombus in rats: Pathological and immunological studies

**DOI:** 10.1002/cre2.215

**Published:** 2019-07-25

**Authors:** Takehisa Iwai, Yoshiki Matsui, Kaori Homma, Tamiko Takemura, Mutsunori Fujiwara, Norio Aoyama, Hiroki Sato, Yuichi Izumi

**Affiliations:** ^1^ Division of Vascular Surgery and Collagen Disease Medicine Tsukuba Vascular Center Ibaraki Japan; ^2^ Section of Pathology Japanese Red‐Cross Medical Center Tokyo Japan; ^3^ Department of Periodontology Kanagawa Dental University Kanagawa Japan; ^4^ Department of Periodontology Tokyo Medical and Dental University Tokyo Japan

**Keywords:** inflammatory thrombus, oral bacterial injection, *Porphyromonas gingivalis*, regular immunological changes

## Abstract

**Objectives:**

Our study investigated the pathological outcome of experimental thrombi that incorporate oral bacteria.

**Material and methods:**

A small artery and vein in the rats' groins were injected with a solution containing periodontal bacteria *Porphyromonas gingivalis* and followed up for 28 days. In all, 18 limbs of nine male rats (500–650 g) were used for the arterial study, and eight limbs of four rats were used for the veins. Two densities of the bacterial solution and two arterial thicknesses sizes were used in the arterial study. Both proximal and distal arteries and veins were ligated loosely using a monofilament nylon suture before bacterial suspensions or control solutions were injected into the ligated vessels.

**Results:**

After 7, 14–18, and 28 days, the rats were sacrificed. Pathology and immunohistochemistry were performed. All specimens exhibited thrombus formation and an acute inflammation reaction with granulocytes at 7 days and then settled down to chronic fibrous change with plasma cells or macrophages at 28 days in the arterial thrombus. CD3 (Pan T‐cells), CD79a (Pan B cells in the rats), and IgG were observed in the process of the healing of the arterial thrombus. Venous changes showed relatively clear recanalization that appeared at 7 days, which is slightly different from the artery. Granulocytes were present from 7 to 28 days.

**Conclusions:**

Periodontal bacteria act as an inflammatory core in the vessels, but not as an infectious agent, in our experiments, because of their low ability to invade tissues.

## 1 INTRODUCTION

Bacteria associated with chronic periodontitis have been associated with a number of other conditions including atherosclerosis(Li, Messas, Batista, Levine, & Amar, 2002), vasculitis(Iwai et al., 2005), cerebrovascular disease(Wu et al., 2000), pre‐term low‐birth‐weight babies(Vergnes & Sixou, 2007) and rheumatoid arthritis(Mikuls et al., 2014). The relationships, between periodontitis‐associated bacteria and other conditions, have been identified mainly through statistical analysis. The purpose of our study is to investigate the potential mechanisms through which periodontitis‐associated bacteria might also be associated with some diseases of blood vessels. We wish to investigate any toxic effects and pattern of action of periodontitis‐associated bacteria on the blood vessels.

After periodontal bacteria enter the blood stream, they are transported from around the teeth into the body through venous and lymphatic pathways. In the blood stream, the bacteria can survive within platelets (Li et al., 2008) or monocytes (Suwatanapongehed, Surarit, Srisatjaluk, & Offenbacher, 2010), possibly for several hours. Within the blood stream, periodontal bacteria can cause microemboli or adhesion to the luminal surface of blood vessels. The major difference between proliferative bacteria, such as *Staphylococcus aureus*, and periodontal bacteria is the lack of aneurysmal formation (Hsu, Tsay, Wang, & Chu, 2002) or production of infectious arteritis by oral bacteria. The presence of periodontal bacteria within blood vessels has been confirmed by the detection of the DNA of the bacteria (Ashimoto, Chen, Bakker, & Slots, 1996). Bacteria that survive within platelets not only form microemboli but also release cytokines or induce chronic inflammation (Yamazaki et al., 1997).

It is difficult to observe the pathological characteristics of the activities of oral bacteria within the human circulatory system, or in experimental rats, because any reaction to the bacteria occurs in small vessels (Kubota et al., 2008). An experimental model in rats used an intravenous continuous‐infusion pump to demonstrate a high count of bacterial DNA in peripheral areas such as the foot (Kubota et al., 2008). Microscopic observation is limited in such areas. Angiographic changes observed in peripheral vessels also showed that it is difficult to investigate pathological changes within these types of vessels.

Vessels in the groin were thought to be the smallest ones that could be used in experiments as model vessels without distal tissue loss. We ligated sections of these vessels and injected suspensions of the periodontitis associated bacterium *Porphyromanas gingivalis* into such sites. This experimental system induces formation of intravascular thrombi containing the bacterium. Our study describes the microscopic and microbiological investigation of experimental thrombi produced in rats through the injection of a suspension of *P.* *gingivalis* into small arteries and veins.

## 2 MATERIALS AND METHODS

**Figure 1:**
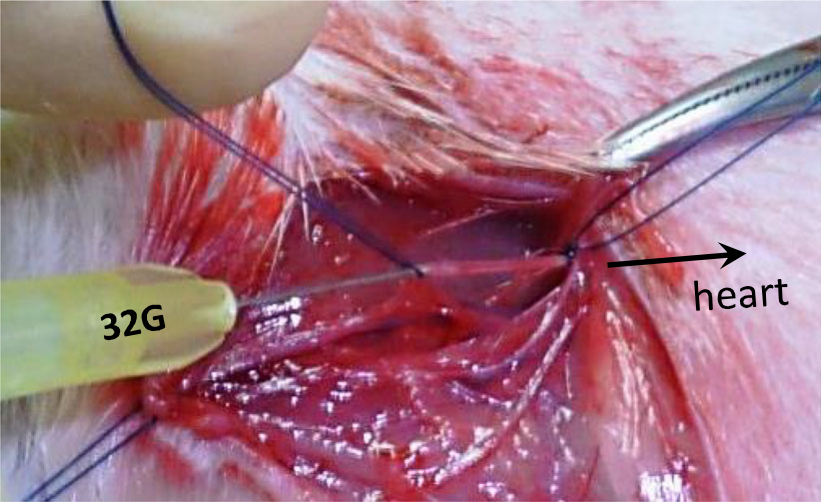
Arterial puncture of the bacterial solution in a rat and the technical method of the injection into the artery and vein, respectively. This shows the small needle (32‐gauge) injection of the Porphyromonas gingivalis bacterial solution in the loosely ligated vessel lumen

In 13 SD rats, 26 vessels were studied. The body weight was about 500–650 g. *P.* *gingivalis* bacteria were chosen as they are representative of periodontitis‐associated bacteria and are easy to handle. Pure cultured *P.* *gingivalis* was obtained from the Laboratory of Periodontal Department of Tokyo Medical and Dental University. Under general anesthesia, an artery about 10 mm long in the groin area was explored using a sterile technique. The rat groin arteries (18 vessels of nine rats) were loosely ligated using 4‐0 nylon monofilament suture proximally and distally (Figure 1). The sausage‐like vessels were injected with two different densities of bacterial solution (low density 1 × 10^7^ colony‐forming units (CFU)/ml and high density 1 × 10^8^ CFU/ml of *P.* *gingivalis* bacteria) and phosphate buffer saline (PBS) as a control without bacteria. A 32‐gauge injection needle was used. The sign of a good injection was the expansion of the arterial lumen. A thick artery was about 1 mm in diameter and a thin one (seven vessels) was 0.6 mm. The injected solution volume was about 0.3 and 0.1 ml. The veins (eight vessels of four rats) were treated as same manner. After 7, 14–18, and 28 days, the vessels were removed from the sacrificed animals.

Specimen fixation was conducted using a 10% formalin solution or a 4% paraformaldehyde phosphate buffer solution, followed by embedding in paraffin. Hematoxylin Eosin and Elastica van Gieson staining were applied. The smooth muscle cell staining was done separately. Components of the blood cells of all the vessel thrombi, including the vessel walls, were analyzed as much as possible, including red blood cells (RBC), granulocytes, usual lymphocytes, plasm cells, macrophages, platelets, giant cells, elastic fibers, hemosiderin, fibrin and recanalization, including their quantitative grades (+ less than 10 cells to +++21 cells and over, or +few to +++ rich for a noncell material). The internal elastic lamina (IEL) was classified by disruption, shrinkage, and reduplication. Immunohistochemical staining was applied in 15 arteries, and CD3 (Pan T‐cell), CD79a (Pan B‐cell in the rat), and IgG were demonstrated. For the staining from paraffin sections of arterial tissue, the preparation was carried out by New Histo. Science Laboratory Co. Ltd., Tokyo, Japan (http://www.hislabo.co.jp/Tel +81‐428‐74‐4741).

## 3 RESULTS

**Figure 2:**
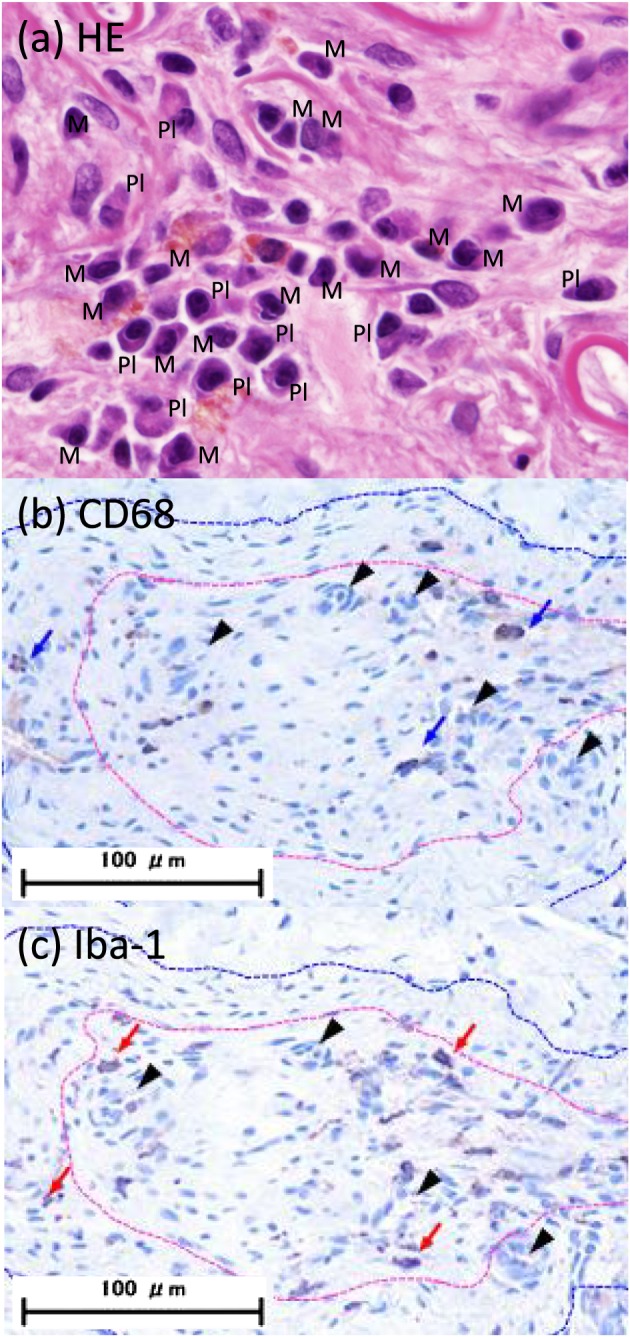
Monocytes/macrophages staining in the specimen, which is 28 days, thick size and low density solution injected‐specimen. HE staining (a) and immunochemical stainings (b,c). (a) M:monocyte/macrophage, Pl: plasma cell. (b) CD68 positive cells ++ (11–29 cells), blue arrows indicate the representative cells, black arrows: capillaries. (c) Iba‐1 positive cells ++, red arrows indicate the representative cells

**Figure 3:**
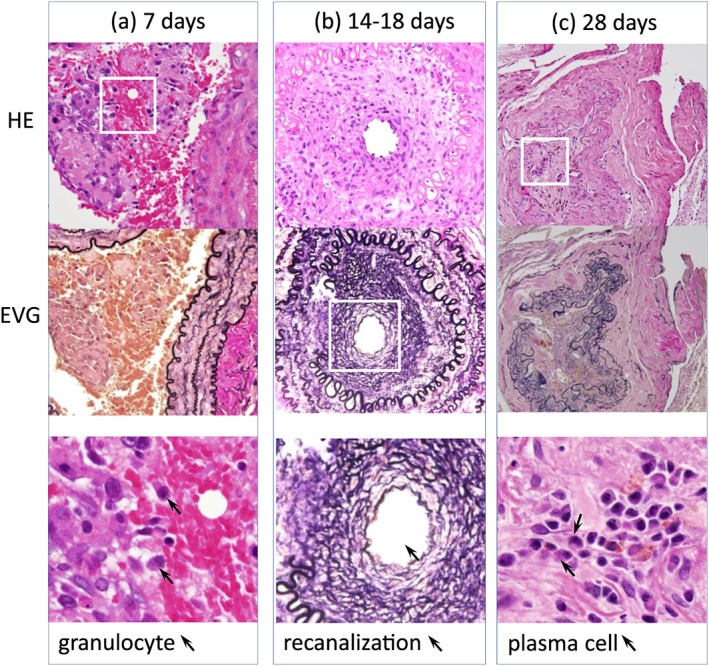
Pathological serial changes after injection of Porphyromonas gingivalis (1 × 10^8^ colony‐forming units [CFU]/ml) of the artery. The two densities are compared in Table 1. (a) 7 days (1 × 10^8^ CFU/ml) after injection. In the thrombus, many red cells, moderate granulocytes, a few lymphocytes, macrophages, and spindle cells are shown. Internal elastic lamina (IEL) is intact. In the media, granulocytes and edema are present (above HE, low EVG stain × 400). (b) 14‐18 days (1 × 10^8^ CFU/ml) after injection. In the thrombus, granulocytes disappeared and recanalization appeared. IEL shows disruption, shrinkage, and reduplication. Edematous media and outer elastic lamia disruption are present (EVG × 100, HE x100). (c) 28 days [1 × 10^8^ CFU/ml]) after injection. Lymphocytes and plasma cells are present in the thrombus. Elastic fibers are numerous. IEL shows shrinkage. The media reveals the minimal changes (EVG and HE × 100)

**Figure 4:**
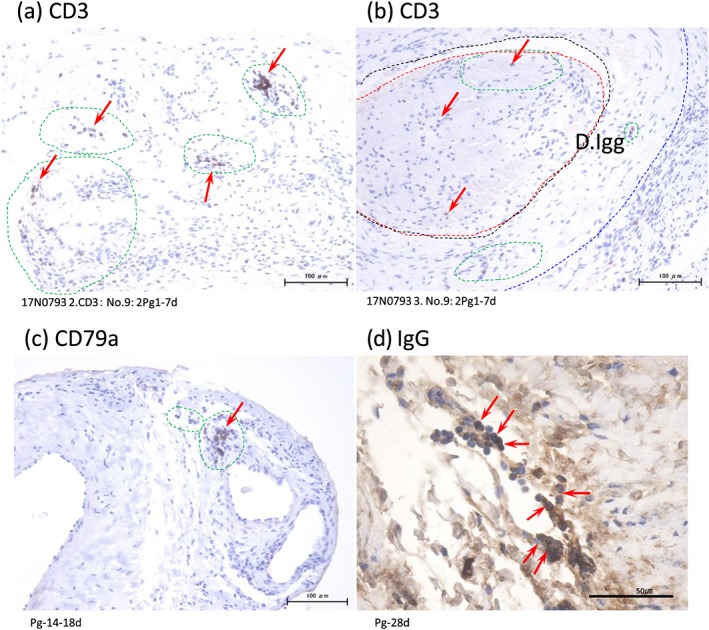
Immunohistochemistry of the specimen. (a) CD3 (Pan B‐cell). Porphyromonas gingivalis bacteria injection after 7 days. More than 30 positive cells around the artery. (b) CD3 (Pan B‐cell). P. gingivalis bacteria injection after 7 days. At least eight positive cells in the thrombus. (c) CD79a (Pan B‐cell). P. gingivalis bacteria injection after 7 days. About 15 positive cells around the artery. (d) IgG in the lymphatic cells of P. gingivalis injection 28 days (arrows)

One control rat died, but there was no wound infection and no limb necrosis in any of the cases. The vein cases showed no edematous changes in the legs. The results showed the ligation site was adequate. Animal care was satisfied by obtaining permission from the animal laboratory. The rats' ligation site was the external iliac artery, which does not cause limb necrosis, even in human cases. The vein site was the external iliac vein, which does not usually cause leg edema. Thrombus formation was observed in all specimens, including the control group. Granulocytes (neutrophils), lymph cells, disruption of the IEL, and recanalization were seen 7 days after injection. Granulocytes appeared ++ (10–19cells/thrombus) in the early phase to zero in the late phase. This is a clear difference without statistical analysis. Same response was observed for plasma cells in the early phase zero to + or ++ in the late phase. The difference between the densities is not so clearly demonstrated as far as the number of granulocytes at 7 days and plasmas cells at 28 days, and the difference between the thicknesses is apparent in the numbers of plasma cells at 28 days and elastic fiber or IEL changes. At 14–18 days, the granulocytes disappeared, and the macrophages (Figure 2), plasma cells (B cell lineage), and fibrous cells increased. The 28‐day specimens showed no inflammatory cells and advanced IEL disruption, and clear recanalization was seen (Figure 3). Control using PBS showed no granulocytes or IEL disruption in the early stages at 7 days or 14–18 days, and no plasma cells in the late stage at 28 days after injection. In the media, granulocytes were + or ++ in the early phase and almost zero in the late phase. Only an edematous condition was observed in all the phases (Table 1). Immunohistochemistry staining showed the increase of the CD3 (Pan T cells), CD79a (Pan B cells in the rat), and IgG in all time periods. IgG in the cells was observed throughout the study without a characteristic location (Figure 4). The control study of the immunochemical study showed only (+) changes compared (+) with (++) changes in the *P.* *gingivalis* injected specimens (Table 2).

**Table 1:**
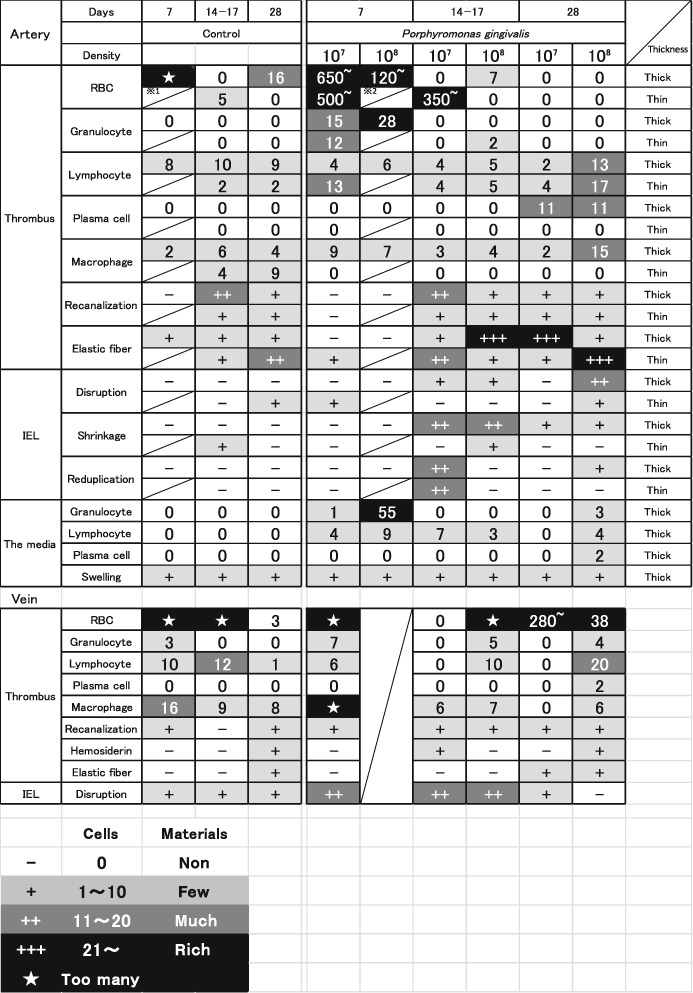
HE and EVG staining results of the artery and vein

*Note.* Control: PBS (phosphate buffered saline). 10 (Suwatanapongehed et al., 2010): 1x10^7^ CFU, 10 (Hsu et al., 2002): 1x10^8^ CFU, Quantitative count criteria: Real counts for cells. Blood cells (RBC (red blood cells), granulocytes, lymphocytes, plasma cells, macrophages). Less than 10 cells + (slight gray zone), 11–20 cells ++ (gray zone), 21 cells‐ +++ (black zone). Elastic fiber, internal elastic lamina (IEL), (disruption, shrinkage, reduplication), and recanalization's classification: non, few +, moderate ++, rich +++.

Abbreviations: CFU: Colony‐forming units/ml, density of bacteria. ※1: dead, ※2: takeout mistake.

**Table 2:**
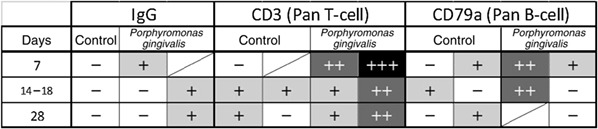
Immunohistochemistry of the arterial specimen

*Note.* Count criteria: positive cells (in the entire arterial specimen, except the granulation tissue). Less than 10 cells +, 11‐29 cells ++, 30 cells +++.

**Figure 5:**
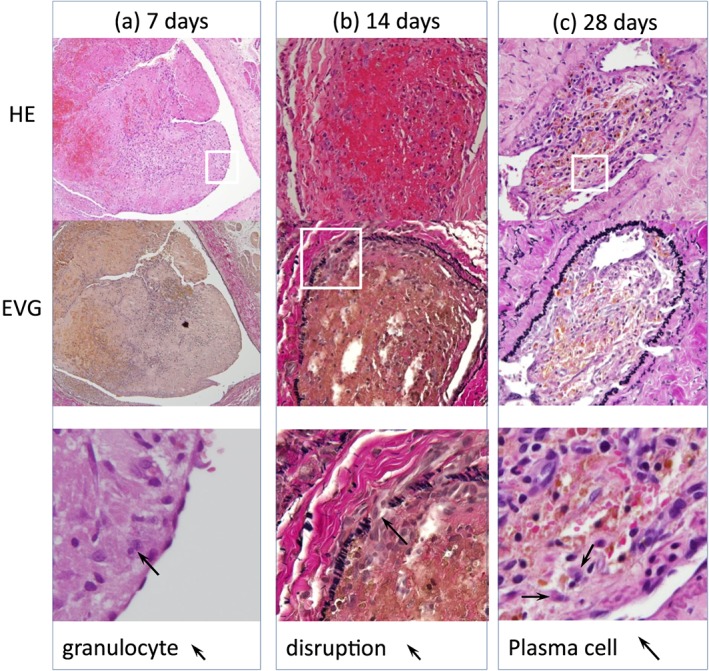
Pathological serial changes after injection of Porphyromonas gingivalis bacteria in the rat vein. (a) 7 days after the 1 × 10^7^ colony‐forming units (CFU)/ml injection of Pg. Fresh thrombus contains red blood cells, macrophages, slight lymphocytes, and granulocytes. Internal elastic lamina (IEL) shows disruption. HE and EVG stain × 100. (b) 14 days after the 1 × 10^8^ CFU/ml injection of P. gingivalis. In the thrombus, rich red blood cells, few granulocytes, macrophages, and spindle cells. IEL shows disruption. HE stain × 200, EVG stain × 400. (c) 28 days after the injection. In the partial thrombus, there are few granulocytes, plasma cells, lymphocytes, macrophages and spindle cells. IEL has small changes

The venous changes were relatively clear recanalization at 7 days, and presence of granulocytes from 7 to 28 days. Other changes include a significant amount of hemosiderin in the thrombus throughout the study. Disruption of IEL was observed in the control group, but there was more disruption in the *P.* *gingivalis* injected specimens. (Figure 5).

## 4 DISCUSSION

Early development of recanalization in the arterial thrombus and in the venous thrombus models suggests ligation with two sutures was secure although it permitted some vascular flow through the area of experimental clots. In our series, thrombus formation occurred in 100% of the specimens. Our method is useful compared with other methods, such as making stenosis or reoperation for ligation removal. The pathological findings did not show the calcification or microabscess formation, which are seen in one type of vasculitis (Tanaka, 1998).

Histology, using H&E and Elastica van Gieson staining, indicated aseptic inflammation at the experimental site. This inflammation diminished at around 28 days. The result suggests a transition from the acute phase of inflammation to a chronic phase within 28 days. Immune responses of model mice infected with *P.* *gingivalis* reach their maximum at around 3–5 weeks (Harada, 2002). During experimental periodontitis associated with *P.* *gingivalis*, in a previous study (Harada, 2002), staining for IgG slowly increased from 3 weeks and reached its maximum at 5–7 weeks, and CD3 staining in an area of thrombus reached its maximum at 3 weeks after infection. The findings in the previous study(Harada, 2002), which are similar to our result, suggest that the expected pattern of inflammation associated with *P.* *gingivalis* was observed in the injected blood vessels in our experiments.

Nearly 1,000 different species of bacteria inhabit the gingival crevices or pockets associated with chronic periodontitis. Many of these periodontitis‐associated bacteria are anaerobic gram‐stain negative bacteria or spirochetes (Offenbacher, Barros, & Beck, 2008). These bacteria inhabit periodontal pockets associated with periodontal bone loss (Offenbacher et al., 2008). As shown previously, *P. gingivalis* bacteria are found in periodontal pockets and are also associated with several other important diseases (Iwai et al., 2005; Li et al., 2002; Mikuls et al., 2014; Vergnes & Sixou, 2007; Wu et al., 2000). In moderate periodontitis, teeth brushing, dental flossing, or tooth extraction will produce bacteremia (Carroll & Sebor, 1980; Lockhart et al., 2008) and the continuous intravascular inflammatory reaction we demonstrated previously in small arterial and venous experimental models (Kubota et al., 2008).

Other periodontal bacteria such as *Treponema denticola* might enhance vascular damage induced by *P.* *gingivalis.* *P.* *gingivalis* is also known to be associated with monocytes (Suwatanapongehed et al., 2010), which act directly on the endothelial lumen. This phenomenon may promote atherosclerotic changes in the midsized artery (Suwatanapongehed et al., 2010).

In many cases, cigarette smoking is strongly related to the worsening of the disease. Nicotine, which is associated with periodontitis (Barbour et al., 1997), suppresses the immune system. One component of treatment for periodontitis is smoking cessation.

The experimental thrombus in veins is similar to the thrombusof the phlebitis migrans that has been shown to contain *P.* *gingivalis* bacteria (Iwai et al., 2012). Bacteria traversing capillaries may cause small vein phlebitis. Venous inflammatory change caused by experimental injection of *P.* *gingivalis* in our study help to support observations of the presence of DNA from *P.* *gingivalis* in varicose veins (Kurihara et al., 2007). It would be useful to perform immunohistochemical studies on venous specimens to obtain additional information. Immunological reactions in arterial thrombi and surrounded areas that are associated with the presence of DNA from *P.* *gingivalis* are not well understood. Forms of vasculitis, such as Buerger's disease, are strongly related to T‐cell mediated or B‐cell mediated immune systems (Kobayashi, Ito, Nakagawa, Nishikimi, & Nimura, 1999; Lee, Seo, Sumpio, & Kim, 2003). In Buerger's disease, the causative antigen or type of inflammation is not known, but the condition is associated with active cigarette smoking. Chen et al. (2007) reported that vasculitis patients had significantly higher IgG titers against *P. gingivalis* and *Aggregatibacter actinomycetemconcomitant* than unaffected individuals.

The thrombosis model that employed *P.* *gingivali*s, demonstrated pathological and immunohistochemical aspects of experimental thrombus formation. *P.* *gingivalis* injected vessels showed an acute inflammatory reaction followed by chronic fibrous changes in only 28 days. Immunohistochemistry showed the expected inflammatory changes induced by noninvasive bacteria. These immunohistochemistry changes seemed to be a natural immunological response. We did not observe notable differences in the pathological changes when we used either the lower or higher doses of bacteria. Further investigation with this experimental model will provide more information about vascular diseases associated with the bacteria found in chronic periodontitis.
